# (*E*)-17β,19-Epoxy­methano-17,23,24-tridemethyl-4-nor-5β,18α-olean-3-one oxime

**DOI:** 10.1107/S1600536809016675

**Published:** 2009-05-14

**Authors:** Anna Froelich, Oxana B. Kazakova, Genrikh Tolstikov, Andrzej K. Gzella

**Affiliations:** a Department of Organic Chemistry, Poznan University of Medical Sciences, ul. Grunwaldzka 6, 60-780 Poznań, Poland; b Institute of Organic Chemistry, Ufa Research Center of the Russian Academy of Sciences, 71, prosp. Oktyabrya, 450054 Ufa, Russian Federation

## Abstract

In the penta­cyclic triterpenoide skeleton of the title mol­ecule, C_27_H_43_NO_2_ [systematic name: (3*E*,3a*S*,5a*R*,5b*R*,7a*R*,11*R*,11a*R*,11b*R*,13a*R*,13b*R*)-5a,5b,10,10,13b-penta­methyl­icosa­hydro-1*H*-11,7a-(epoxy­methano)cyclo­penta­[*a*]chrysen-3-one oxime], the five-membered ring *A* has an envelope conformation, while the six-membered rings *B*–*E* adopt chair conformations. Rings *A* and *B* are *cis*-fused. The hydroximino group has an *E* configuration. Strong inter­molecular O—H⋯O hydrogen bonds link the mol­ecules into helical chains.

## Related literature

For the syntheses of related compounds, see: Medvedeva *et al.* (2004 ▶, 2006 ▶);  Gzella *et al.* (1997 ▶, 1998 ▶); Zaprutko (1995 ▶, 1997 ▶). For a description of the Cambridge Structural Database, see: Allen (2002 ▶). For puckering parameters, see: Cremer & Pople (1975 ▶); Spek (2009 ▶).
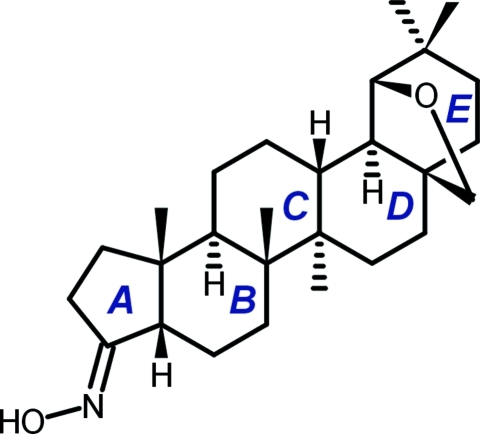


## Experimental

### 

#### Crystal data

C_27_H_43_NO_2_
*M* *_r_* = 413.62
Orthorhombic, 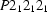

*a* = 12.5887 (16) Å
*b* = 13.2550 (11) Å
*c* = 14.5355 (12) Å
*V* = 2425.4 (4) Å^3^
*Z* = 4
Cu *K*α radiation
μ = 0.53 mm^−1^
*T* = 293 K
0.40 × 0.22 × 0.13 mm

#### Data collection

Kuma Diffraction KM-4 diffractometer
Absorption correction: none
4994 measured reflections
2610 independent reflections
2240 reflections with *I* > 2σ(*I*)
*R*_int_ = 0.037
3 standard reflections every 100 reflections intensity decay: 2.3%

#### Refinement

*R*[*F* ^2^ > 2σ(*F* ^2^)] = 0.031
*wR*(*F* ^2^) = 0.094
*S* = 1.06
2610 reflections
281 parameters
H atoms treated by a mixture of independent and constrained refinement
Δρ_max_ = 0.12 e Å^−3^
Δρ_min_ = −0.12 e Å^−3^

### 

Data collection: *KM-4 Software* (Kuma Diffraction, 1996 ▶); cell refinement: *KM-4 Software*; data reduction: *KM-4 Software*; program(s) used to solve structure: *SHELXS97* (Sheldrick, 2008 ▶); program(s) used to refine structure: *SHELXL97* (Sheldrick, 2008 ▶); molecular graphics: *ORTEP-3 for Windows* (Farrugia, 1997 ▶); software used to prepare material for publication: *WinGX* (Farrugia, 1999 ▶).

## Supplementary Material

Crystal structure: contains datablocks I, global. DOI: 10.1107/S1600536809016675/fb2138sup1.cif

Structure factors: contains datablocks I. DOI: 10.1107/S1600536809016675/fb2138Isup2.hkl

Additional supplementary materials:  crystallographic information; 3D view; checkCIF report

## Figures and Tables

**Table 1:** Hydrogen-bond geometry (Å, °)

*D*—H⋯*A*	*D*—H	H⋯*A*	*D*⋯*A*	*D*—H⋯*A*
O1—H1⋯O2^i^	0.87 (3)	1.93 (3)	2.782 (2)	164 (3)

Symmetry code: (i) 
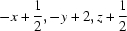
.

**Fig. 1.**
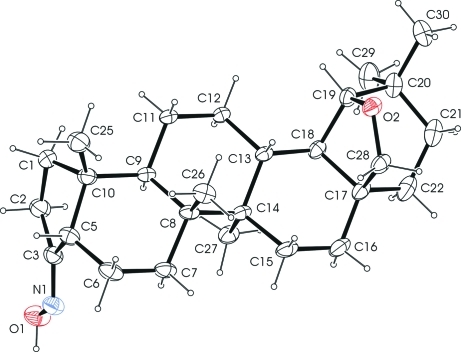
The title molecule showing the atomic labeling scheme. The displacement ellipsoids are shown at the 30% probability level.

**Fig. 2.**
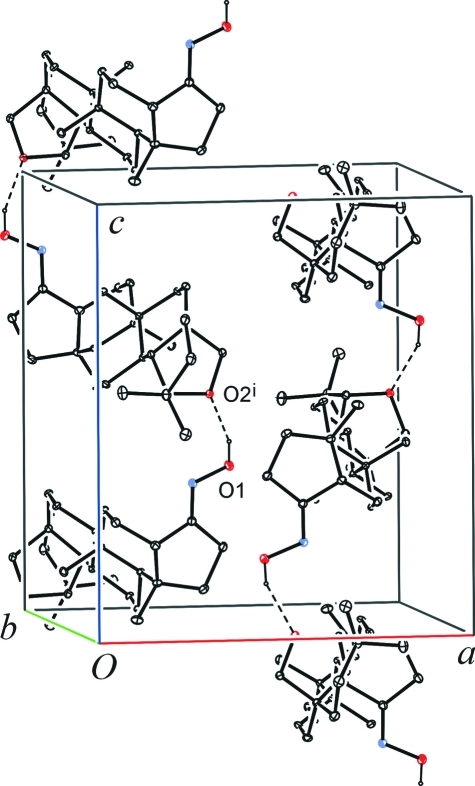
The hydrogen bonding (dashed lines) in the title structure. Symmetry code: (i) 0.5-x, 2-y, 1/2+z. The H atoms not involved in hydrogen bonds have been omitted for clarity.

## supplementary crystallographic information

### Comment

One of the steps in our synthesis of the title compound, (*E*)-17β,19-epoxymethano-17,23,24-tridemethyl-4-nor-5β,18α-olean-3-one oxime (Scheme 1; Fig. 3 - (IV)), from allobetulin (Fig. 3 - (I)) involved ozonolysis of the intermediate *δ*-apoallobetulin, Fig. 3 - (II), to give 17β,19-epoxymethano-17,23,24-tridemethyl-4-nor-5β,18α-olean-3-one (Fig. 3 - (III)) as the transformation product with a *cis*-junction between the *A*/*B* rings. It should be mentioned that synthetic conversions to new derivatives with altered junction of *A*/*B* rings are rarely observed in triterpenoids of the oleanane group [Zaprutko (1995, 1997); Gzella *et al.* (1997); Gzella *et al.* (1998); Medvedeva *et al.* (2004)].

The X-ray structure determination of the title compound was carried out in order to confirm its spatial structure that had been proposed on the basis of spectroscopic data by Medvedeva *et al.* (2004).

The results obtained for the title compound confirm the *cis*-junction of *A*/*B* rings. The corresponding interplanar angle between the least-squares planes of the *A*/*B* rings is 71.85 (8)°. The H atom at the C5 asymmetric centre exhibits β-orientation and occupies a pseudo-axial position with respect to the *A* ring and an equatorial position to the *B* ring [the angles of the H5—C5 bond vector to the Cremer & Pople *A* and *B* ring plane normals are 9.60 (9) and 64.05 (7)°, respectively (Cremer & Pople, 1975; Spek, 2009)]. The torsion angle H5—C5—C10—C25 of 38° reveals a halfway conformation between synperiplanar and synclinal for bonds H5—C5 and C10—C25.

In the molecule the six-membered rings *B*–*E* of the pentacyclic ring system are *trans*-fused as in allobetuline. The dihedral angles between the least-squares planes of these rings are *B*/*C* 7.09 (10), *C*/*D* 0.80 (10), *D*/*E* 14.95 (9)°.

In the title structure, each of the six-membered rings *B*–*E* has a differently distorted chair conformation, whereas both five-membered rings, *i.e.* the carbocyclic ring *A* and the heterocyclic ring C17\C18\C19\O2\C28 including epoxymethylene group, assume envelope conformations. The respective puckering parameters (Cremer & Pople, 1975) are *Q* = 0.373 (2) Å, Φ = 147.1 (4)° and *Q* = 0.470 (2)Å, Φ = 253.2 (2)°.

The hydroximino function in C3 position has the *E* configuration. The value of the torsion angle O1—N1—C3—C5 is -179.80 (18)°.

The molecular packing is stabilized by O1—H···O2^i^ hydrogen bonds (Tab. 1). The hydroxyl hydrogen is donated to the remote-ring epoxy O atom from the neighbour molecule. These hydrogen bonds link the molecules into helical chains which proceed in the *c* direction (Fig. 2).

### Experimental

The title compound was obtained according to the procedure described by Medvedeva *et al.* (2004). Single colourless needle-crystals suitable for analysis were grown from ethanol by slow evaporation at room temperature.

### Refinement

All the hydrogens were discernible in the difference electron density map. Except for the hydroxyl H atom that was refined freely the remaining hydrogens were situated into the idealized positions and were refined within the riding model approximation: C_methyl_—H = 0.96, C_methylene_—H 0.97, C_methine_= 0.98 Å. *U*_iso_(H) = 1.2 *U*_eq_C_methylene_/C_methine_; *U*_iso_(H) = 1.5*U*_eq_(C_methyl_). The methyl group was allowed to rotate during refinement. The absolute configuration of the title structure is known by reference to the known chirality of the enantiopure allobetulin employed as the initial reagent used in the synthesis as well as to the chirality of the other oleanane derivatives [see: CSD, Cambridge; Allen (2002)].

### Crystal data

**Table:**

C_27_H_43_NO_2_	*D*_x_ = 1.133 Mg m^−^^3^
*M**_r_* = 413.62	Melting point = 468–470 K
Orthorhombic, *P*2_1_2_1_2_1_	Cu *K*α radiation, λ = 1.54178 Å
Hall symbol: P 2ac 2ab	Cell parameters from 54 reflections
*a* = 12.5887 (16) Å	θ = 14.5–28.5°
*b* = 13.2550 (11) Å	µ = 0.53 mm^−^^1^
*c* = 14.5355 (12) Å	*T* = 293 K
*V* = 2425.4 (4) Å^3^	Needle, colourless
*Z* = 4	0.40 × 0.22 × 0.13 mm
*F*(000) = 912	

### Data collection

**Table:**

Kuma Diffraction KM-4 diffractometer	*R*_int_ = 0.037
Radiation source: fine-focus sealed tube	θ_max_ = 70.1°, θ_min_ = 4.5°
graphite	*h* = −15→15
ω/2θ scans	*k* = 0→16
4994 measured reflections	*l* = 0→17
2610 independent reflections	3 standard reflections every 100 reflections
2240 reflections with *I* > 2σ(*I*)	intensity decay: 2.3%

### Refinement

**Table:**

Refinement on *F*^2^	Secondary atom site location: difference Fourier map
Least-squares matrix: full	Hydrogen site location: difference Fourier map
*R*[*F*^2^ > 2σ(*F*^2^)] = 0.031	H atoms treated by a mixture of independent and constrained refinement
*wR*(*F*^2^) = 0.094	*w* = 1/[σ^2^(*F*_o_^2^) + (0.0523*P*)^2^ + 0.1689*P*] where *P* = (*F*_o_^2^ + 2*F*_c_^2^)/3
*S* = 1.06	(Δ/σ)_max_ < 0.001
2610 reflections	Δρ_max_ = 0.12 e Å^−^^3^
281 parameters	Δρ_min_ = −0.12 e Å^−^^3^
0 restraints	Extinction correction: *SHELXL*, Fc^*^=kFc[1+0.001xFc^2^λ^3^/sin(2θ)]^-1/4^
163 constraints	Extinction coefficient: 0.0011 (2)
Primary atom site location: structure-invariant direct methods	

### Special details

**Table:**

Geometry. All esds (except the esd in the dihedral angle between two l.s. planes) are estimated using the full covariance matrix. The cell esds are taken into account individually in the estimation of esds in distances, angles and torsion angles; correlations between esds in cell parameters are only used when they are defined by crystal symmetry. An approximate (isotropic) treatment of cell esds is used for estimating esds involving l.s. planes.
Refinement. Refinement of *F*^2^ against ALL reflections. The weighted *R*-factor *wR* and goodness of fit *S* are based on *F*^2^, conventional *R*-factors *R* are based on *F*, with *F* set to zero for negative *F*^2^. The threshold expression of *F*^2^ > σ(*F*^2^) is used only for calculating *R*-factors(gt) *etc*. and is not relevant to the choice of reflections for refinement. *R*-factors based on *F*^2^ are statistically about twice as large as those based on *F*, and *R*- factors based on ALL data will be even larger.

### Fractional atomic coordinates and isotropic or equivalent isotropic displacement parameters (Å^2^)

**Table:**

	*x*	*y*	*z*	*U*_iso_*/*U*_eq_	
O1	0.50319 (13)	0.77700 (17)	0.33765 (13)	0.0806 (5)	
H1	0.491 (2)	0.762 (2)	0.395 (2)	0.100 (10)*	
O2	0.06258 (10)	1.29692 (10)	0.00744 (9)	0.0561 (3)	
N1	0.40209 (14)	0.75851 (15)	0.29992 (12)	0.0620 (5)	
C1	0.43803 (18)	0.79829 (17)	0.05510 (15)	0.0631 (5)	
H1A	0.4512	0.7343	0.0248	0.076*	
H1B	0.4666	0.8519	0.0170	0.076*	
C2	0.49002 (17)	0.7997 (2)	0.15037 (16)	0.0680 (6)	
H2A	0.5484	0.7521	0.1537	0.082*	
H2B	0.5162	0.8666	0.1654	0.082*	
C3	0.40138 (16)	0.76952 (16)	0.21323 (14)	0.0561 (5)	
C5	0.30026 (17)	0.75334 (15)	0.16039 (14)	0.0576 (5)	
H5	0.2966	0.6817	0.1441	0.069*	
C6	0.19970 (17)	0.77938 (18)	0.21268 (15)	0.0658 (6)	
H6A	0.1384	0.7587	0.1769	0.079*	
H6B	0.1985	0.7425	0.2703	0.079*	
C7	0.19268 (16)	0.89107 (17)	0.23218 (13)	0.0591 (5)	
H7A	0.2513	0.9103	0.2718	0.071*	
H7B	0.1272	0.9046	0.2652	0.071*	
C8	0.19556 (13)	0.95683 (15)	0.14448 (11)	0.0455 (4)	
C9	0.29594 (14)	0.92829 (13)	0.08732 (11)	0.0427 (4)	
H9	0.3562	0.9514	0.1245	0.051*	
C10	0.31736 (16)	0.81371 (14)	0.07022 (13)	0.0526 (4)	
C11	0.30140 (16)	0.99102 (13)	−0.00084 (11)	0.0475 (4)	
H11A	0.3657	0.9740	−0.0341	0.057*	
H11B	0.2413	0.9743	−0.0397	0.057*	
C12	0.30058 (14)	1.10370 (13)	0.01892 (12)	0.0457 (4)	
H12A	0.2966	1.1402	−0.0388	0.055*	
H12B	0.3667	1.1221	0.0488	0.055*	
C13	0.20788 (13)	1.13561 (13)	0.08002 (11)	0.0415 (4)	
H13	0.1426	1.1200	0.0462	0.050*	
C14	0.20485 (13)	1.07304 (15)	0.17054 (10)	0.0456 (4)	
C15	0.10814 (16)	1.10874 (19)	0.22730 (13)	0.0629 (6)	
H15A	0.0437	1.0922	0.1940	0.075*	
H15B	0.1067	1.0722	0.2851	0.075*	
C16	0.10920 (18)	1.2219 (2)	0.24743 (13)	0.0690 (6)	
H16A	0.0434	1.2399	0.2781	0.083*	
H16B	0.1671	1.2365	0.2894	0.083*	
C17	0.12164 (15)	1.28729 (17)	0.16219 (13)	0.0558 (5)	
C18	0.20859 (14)	1.24982 (14)	0.09614 (12)	0.0476 (4)	
H18	0.2786	1.2714	0.1180	0.057*	
C19	0.17728 (14)	1.31005 (14)	0.01104 (14)	0.0503 (4)	
H19	0.2103	1.2809	−0.0439	0.060*	
C20	0.20282 (19)	1.42351 (16)	0.01733 (18)	0.0691 (6)	
C21	0.1451 (2)	1.46632 (19)	0.1015 (2)	0.0814 (8)	
H21A	0.1774	1.5304	0.1177	0.098*	
H21B	0.0718	1.4794	0.0849	0.098*	
C22	0.14688 (19)	1.3980 (2)	0.18554 (18)	0.0773 (7)	
H22A	0.0954	1.4224	0.2299	0.093*	
H22B	0.2165	1.4016	0.2139	0.093*	
C25	0.2534 (2)	0.76839 (18)	−0.00933 (16)	0.0742 (7)	
H25A	0.2774	0.7008	−0.0209	0.111*	
H25B	0.2634	0.8086	−0.0636	0.111*	
H25C	0.1794	0.7673	0.0066	0.111*	
C26	0.09170 (15)	0.93677 (17)	0.09073 (15)	0.0596 (5)	
H26A	0.0862	0.8661	0.0770	0.089*	
H26B	0.0924	0.9746	0.0344	0.089*	
H26C	0.0320	0.9572	0.1274	0.089*	
C27	0.30490 (15)	1.09487 (17)	0.22921 (12)	0.0561 (5)	
H27A	0.3666	1.0694	0.1981	0.084*	
H27B	0.2981	1.0624	0.2879	0.084*	
H27C	0.3120	1.1663	0.2380	0.084*	
C28	0.02439 (14)	1.28226 (19)	0.09950 (14)	0.0597 (5)	
H28A	−0.0103	1.2172	0.1051	0.072*	
H28B	−0.0261	1.3346	0.1157	0.072*	
C29	0.3229 (2)	1.43864 (18)	0.0252 (2)	0.0896 (9)	
H29A	0.3395	1.5088	0.0175	0.134*	
H29B	0.3581	1.4000	−0.0216	0.134*	
H29C	0.3465	1.4166	0.0847	0.134*	
C30	0.1626 (2)	1.47527 (18)	−0.0701 (2)	0.0876 (8)	
H30A	0.1719	1.5469	−0.0646	0.131*	
H30B	0.0886	1.4603	−0.0784	0.131*	
H30C	0.2020	1.4510	−0.1222	0.131*	

### Atomic displacement parameters (Å^2^)

**Table:**

	*U*^11^	*U*^22^	*U*^33^	*U*^12^	*U*^13^	*U*^23^
O1	0.0541 (8)	0.1201 (15)	0.0675 (10)	0.0019 (9)	−0.0063 (8)	0.0231 (10)
O2	0.0432 (6)	0.0761 (8)	0.0490 (7)	0.0101 (6)	−0.0029 (5)	−0.0061 (7)
N1	0.0510 (9)	0.0778 (12)	0.0573 (9)	0.0013 (8)	−0.0003 (8)	0.0150 (9)
C1	0.0741 (13)	0.0589 (11)	0.0564 (11)	0.0148 (11)	0.0171 (10)	0.0079 (9)
C2	0.0541 (11)	0.0841 (15)	0.0657 (13)	0.0125 (11)	0.0103 (10)	0.0207 (11)
C3	0.0550 (11)	0.0573 (11)	0.0558 (11)	0.0022 (9)	0.0036 (9)	0.0129 (9)
C5	0.0644 (11)	0.0537 (10)	0.0547 (10)	−0.0088 (9)	−0.0001 (10)	0.0128 (9)
C6	0.0531 (11)	0.0814 (14)	0.0629 (12)	−0.0158 (11)	0.0009 (10)	0.0286 (11)
C7	0.0456 (9)	0.0859 (14)	0.0458 (9)	0.0003 (10)	0.0111 (8)	0.0187 (9)
C8	0.0357 (8)	0.0656 (11)	0.0354 (8)	−0.0061 (8)	0.0018 (7)	0.0076 (7)
C9	0.0423 (8)	0.0524 (9)	0.0333 (7)	−0.0045 (7)	0.0039 (7)	0.0028 (7)
C10	0.0635 (11)	0.0504 (9)	0.0440 (9)	−0.0045 (9)	0.0029 (9)	0.0037 (8)
C11	0.0561 (10)	0.0525 (9)	0.0341 (8)	0.0009 (8)	0.0106 (8)	0.0016 (7)
C12	0.0455 (8)	0.0504 (9)	0.0413 (8)	0.0016 (8)	0.0123 (8)	0.0026 (7)
C13	0.0346 (7)	0.0561 (9)	0.0337 (7)	0.0005 (7)	0.0040 (7)	−0.0027 (7)
C14	0.0352 (8)	0.0693 (11)	0.0324 (7)	0.0020 (8)	0.0033 (7)	−0.0014 (7)
C15	0.0471 (10)	0.1020 (17)	0.0395 (9)	0.0119 (10)	0.0126 (8)	0.0045 (10)
C16	0.0566 (11)	0.1094 (18)	0.0408 (9)	0.0257 (12)	0.0037 (9)	−0.0167 (11)
C17	0.0433 (9)	0.0761 (13)	0.0481 (10)	0.0139 (9)	−0.0016 (8)	−0.0173 (10)
C18	0.0354 (8)	0.0603 (11)	0.0472 (9)	0.0046 (7)	0.0007 (8)	−0.0105 (8)
C19	0.0428 (9)	0.0542 (9)	0.0539 (10)	0.0089 (7)	0.0016 (8)	−0.0039 (8)
C20	0.0627 (12)	0.0550 (11)	0.0897 (15)	0.0113 (10)	0.0011 (13)	−0.0083 (11)
C21	0.0754 (15)	0.0661 (14)	0.103 (2)	0.0176 (12)	−0.0056 (14)	−0.0248 (14)
C22	0.0650 (13)	0.0908 (17)	0.0761 (15)	0.0210 (12)	−0.0096 (12)	−0.0400 (14)
C25	0.1048 (18)	0.0607 (14)	0.0572 (12)	−0.0073 (12)	−0.0101 (13)	−0.0062 (11)
C26	0.0426 (9)	0.0751 (13)	0.0609 (11)	−0.0125 (9)	−0.0076 (9)	0.0090 (11)
C27	0.0492 (10)	0.0764 (13)	0.0427 (9)	0.0034 (10)	−0.0086 (8)	−0.0082 (9)
C28	0.0413 (9)	0.0864 (14)	0.0514 (10)	0.0148 (10)	0.0010 (8)	−0.0128 (10)
C29	0.0691 (14)	0.0581 (12)	0.142 (3)	−0.0061 (11)	0.0053 (17)	−0.0063 (15)
C30	0.1021 (19)	0.0549 (12)	0.106 (2)	0.0156 (13)	−0.0037 (17)	0.0115 (13)

### Geometric parameters (Å, °)

**Table:**

O1—N1	1.407 (2)	C14—C27	1.548 (2)
O1—H1	0.88 (3)	C15—C16	1.529 (4)
O2—C28	1.435 (2)	C15—H15A	0.9700
O2—C19	1.455 (2)	C15—H15B	0.9700
N1—C3	1.268 (3)	C16—C17	1.520 (3)
C1—C2	1.532 (3)	C16—H16A	0.9700
C1—C10	1.548 (3)	C16—H16B	0.9700
C1—H1A	0.9700	C17—C28	1.528 (3)
C1—H1B	0.9700	C17—C18	1.538 (2)
C2—C3	1.497 (3)	C17—C22	1.540 (3)
C2—H2A	0.9700	C18—C19	1.524 (3)
C2—H2B	0.9700	C18—H18	0.9800
C3—C5	1.502 (3)	C19—C20	1.541 (3)
C5—C6	1.516 (3)	C19—H19	0.9800
C5—C10	1.551 (3)	C20—C29	1.529 (3)
C5—H5	0.9800	C20—C30	1.531 (4)
C6—C7	1.510 (3)	C20—C21	1.532 (3)
C6—H6A	0.9700	C21—C22	1.520 (4)
C6—H6B	0.9700	C21—H21A	0.9700
C7—C8	1.545 (2)	C21—H21B	0.9700
C7—H7A	0.9700	C22—H22A	0.9700
C7—H7B	0.9700	C22—H22B	0.9700
C8—C26	1.546 (2)	C25—H25A	0.9600
C8—C9	1.559 (2)	C25—H25B	0.9600
C8—C14	1.591 (3)	C25—H25C	0.9600
C9—C11	1.529 (2)	C26—H26A	0.9600
C9—C10	1.562 (3)	C26—H26B	0.9600
C9—H9	0.9800	C26—H26C	0.9600
C10—C25	1.532 (3)	C27—H27A	0.9600
C11—C12	1.521 (2)	C27—H27B	0.9600
C11—H11A	0.9700	C27—H27C	0.9600
C11—H11B	0.9700	C28—H28A	0.9700
C12—C13	1.526 (2)	C28—H28B	0.9700
C12—H12A	0.9700	C29—H29A	0.9600
C12—H12B	0.9700	C29—H29B	0.9600
C13—C18	1.532 (2)	C29—H29C	0.9600
C13—C14	1.556 (2)	C30—H30A	0.9600
C13—H13	0.9800	C30—H30B	0.9600
C14—C15	1.545 (2)	C30—H30C	0.9600
			
N1—O1—H1	100 (2)	C16—C15—H15B	108.9
C28—O2—C19	108.36 (14)	C14—C15—H15B	108.9
C3—N1—O1	111.93 (18)	H15A—C15—H15B	107.7
C2—C1—C10	106.80 (16)	C17—C16—C15	113.84 (16)
C2—C1—H1A	110.4	C17—C16—H16A	108.8
C10—C1—H1A	110.4	C15—C16—H16A	108.8
C2—C1—H1B	110.4	C17—C16—H16B	108.8
C10—C1—H1B	110.4	C15—C16—H16B	108.8
H1A—C1—H1B	108.6	H16A—C16—H16B	107.7
C3—C2—C1	103.30 (18)	C16—C17—C28	112.25 (19)
C3—C2—H2A	111.1	C16—C17—C18	113.46 (16)
C1—C2—H2A	111.1	C28—C17—C18	100.60 (14)
C3—C2—H2B	111.1	C16—C17—C22	112.64 (18)
C1—C2—H2B	111.1	C28—C17—C22	109.79 (18)
H2A—C2—H2B	109.1	C18—C17—C22	107.37 (18)
N1—C3—C2	129.2 (2)	C19—C18—C13	113.07 (14)
N1—C3—C5	119.83 (19)	C19—C18—C17	98.82 (14)
C2—C3—C5	110.97 (17)	C13—C18—C17	114.23 (16)
C3—C5—C6	114.75 (17)	C19—C18—H18	110.1
C3—C5—C10	103.94 (16)	C13—C18—H18	110.1
C6—C5—C10	114.97 (17)	C17—C18—H18	110.1
C3—C5—H5	107.6	O2—C19—C18	102.90 (15)
C6—C5—H5	107.6	O2—C19—C20	109.03 (15)
C10—C5—H5	107.6	C18—C19—C20	114.15 (17)
C7—C6—C5	111.48 (16)	O2—C19—H19	110.2
C7—C6—H6A	109.3	C18—C19—H19	110.2
C5—C6—H6A	109.3	C20—C19—H19	110.2
C7—C6—H6B	109.3	C29—C20—C30	109.3 (2)
C5—C6—H6B	109.3	C29—C20—C21	111.1 (2)
H6A—C6—H6B	108.0	C30—C20—C21	109.90 (19)
C6—C7—C8	113.39 (17)	C29—C20—C19	109.80 (17)
C6—C7—H7A	108.9	C30—C20—C19	108.6 (2)
C8—C7—H7A	108.9	C21—C20—C19	108.1 (2)
C6—C7—H7B	108.9	C22—C21—C20	114.47 (19)
C8—C7—H7B	108.9	C22—C21—H21A	108.6
H7A—C7—H7B	107.7	C20—C21—H21A	108.6
C7—C8—C26	107.47 (15)	C22—C21—H21B	108.6
C7—C8—C9	108.78 (15)	C20—C21—H21B	108.6
C26—C8—C9	111.99 (14)	H21A—C21—H21B	107.6
C7—C8—C14	110.59 (15)	C21—C22—C17	112.79 (19)
C26—C8—C14	110.43 (15)	C21—C22—H22A	109.0
C9—C8—C14	107.59 (14)	C17—C22—H22A	109.0
C11—C9—C8	110.56 (14)	C21—C22—H22B	109.0
C11—C9—C10	112.80 (14)	C17—C22—H22B	109.0
C8—C9—C10	117.42 (14)	H22A—C22—H22B	107.8
C11—C9—H9	104.9	C10—C25—H25A	109.5
C8—C9—H9	104.9	C10—C25—H25B	109.5
C10—C9—H9	104.9	H25A—C25—H25B	109.5
C25—C10—C1	110.89 (19)	C10—C25—H25C	109.5
C25—C10—C5	111.27 (16)	H25A—C25—H25C	109.5
C1—C10—C5	100.84 (16)	H25B—C25—H25C	109.5
C25—C10—C9	114.24 (17)	C8—C26—H26A	109.5
C1—C10—C9	108.67 (16)	C8—C26—H26B	109.5
C5—C10—C9	110.08 (15)	H26A—C26—H26B	109.5
C12—C11—C9	112.05 (14)	C8—C26—H26C	109.5
C12—C11—H11A	109.2	H26A—C26—H26C	109.5
C9—C11—H11A	109.2	H26B—C26—H26C	109.5
C12—C11—H11B	109.2	C14—C27—H27A	109.5
C9—C11—H11B	109.2	C14—C27—H27B	109.5
H11A—C11—H11B	107.9	H27A—C27—H27B	109.5
C11—C12—C13	112.78 (14)	C14—C27—H27C	109.5
C11—C12—H12A	109.0	H27A—C27—H27C	109.5
C13—C12—H12A	109.0	H27B—C27—H27C	109.5
C11—C12—H12B	109.0	O2—C28—C17	106.37 (15)
C13—C12—H12B	109.0	O2—C28—H28A	110.5
H12A—C12—H12B	107.8	C17—C28—H28A	110.5
C12—C13—C18	111.00 (14)	O2—C28—H28B	110.5
C12—C13—C14	111.29 (14)	C17—C28—H28B	110.5
C18—C13—C14	113.42 (14)	H28A—C28—H28B	108.6
C12—C13—H13	106.9	C20—C29—H29A	109.5
C18—C13—H13	106.9	C20—C29—H29B	109.5
C14—C13—H13	106.9	H29A—C29—H29B	109.5
C15—C14—C27	106.84 (14)	C20—C29—H29C	109.5
C15—C14—C13	107.91 (15)	H29A—C29—H29C	109.5
C27—C14—C13	110.26 (15)	H29B—C29—H29C	109.5
C15—C14—C8	111.46 (16)	C20—C30—H30A	109.5
C27—C14—C8	111.84 (15)	C20—C30—H30B	109.5
C13—C14—C8	108.46 (13)	H30A—C30—H30B	109.5
C16—C15—C14	113.33 (18)	C20—C30—H30C	109.5
C16—C15—H15A	108.9	H30A—C30—H30C	109.5
C14—C15—H15A	108.9	H30B—C30—H30C	109.5
			
C10—C1—C2—C3	24.3 (2)	C26—C8—C14—C15	57.27 (18)
O1—N1—C3—C2	−1.0 (4)	C9—C8—C14—C15	179.76 (13)
O1—N1—C3—C5	−179.80 (18)	C7—C8—C14—C27	57.97 (18)
C1—C2—C3—N1	179.6 (2)	C26—C8—C14—C27	176.80 (14)
C1—C2—C3—C5	−1.5 (2)	C9—C8—C14—C27	−60.70 (17)
N1—C3—C5—C6	31.2 (3)	C7—C8—C14—C13	179.78 (14)
C2—C3—C5—C6	−147.80 (19)	C26—C8—C14—C13	−61.40 (17)
N1—C3—C5—C10	157.6 (2)	C9—C8—C14—C13	61.10 (16)
C2—C3—C5—C10	−21.4 (2)	C27—C14—C15—C16	62.1 (2)
C3—C5—C6—C7	66.3 (2)	C13—C14—C15—C16	−56.4 (2)
C10—C5—C6—C7	−54.2 (2)	C8—C14—C15—C16	−175.40 (15)
C5—C6—C7—C8	58.2 (2)	C14—C15—C16—C17	53.3 (2)
C6—C7—C8—C26	67.6 (2)	C15—C16—C17—C28	68.0 (2)
C6—C7—C8—C9	−53.8 (2)	C15—C16—C17—C18	−45.2 (2)
C6—C7—C8—C14	−171.76 (16)	C15—C16—C17—C22	−167.46 (18)
C7—C8—C9—C11	179.45 (15)	C12—C13—C18—C19	71.70 (19)
C26—C8—C9—C11	60.81 (19)	C14—C13—C18—C19	−162.14 (14)
C14—C8—C9—C11	−60.72 (17)	C12—C13—C18—C17	−176.31 (14)
C7—C8—C9—C10	48.1 (2)	C14—C13—C18—C17	−50.1 (2)
C26—C8—C9—C10	−70.6 (2)	C16—C17—C18—C19	164.25 (17)
C14—C8—C9—C10	167.91 (14)	C28—C17—C18—C19	44.17 (19)
C2—C1—C10—C25	−154.75 (18)	C22—C17—C18—C19	−70.62 (18)
C2—C1—C10—C5	−36.8 (2)	C16—C17—C18—C13	43.9 (2)
C2—C1—C10—C9	78.9 (2)	C28—C17—C18—C13	−76.1 (2)
C3—C5—C10—C25	152.20 (18)	C22—C17—C18—C13	169.06 (16)
C6—C5—C10—C25	−81.5 (2)	C28—O2—C19—C18	28.72 (19)
C3—C5—C10—C1	34.5 (2)	C28—O2—C19—C20	−92.8 (2)
C6—C5—C10—C1	160.79 (18)	C13—C18—C19—O2	75.99 (17)
C3—C5—C10—C9	−80.1 (2)	C17—C18—C19—O2	−45.17 (17)
C6—C5—C10—C9	46.1 (2)	C13—C18—C19—C20	−166.02 (16)
C11—C9—C10—C25	−48.7 (2)	C17—C18—C19—C20	72.81 (19)
C8—C9—C10—C25	81.7 (2)	O2—C19—C20—C29	177.7 (2)
C11—C9—C10—C1	75.7 (2)	C18—C19—C20—C29	63.3 (3)
C8—C9—C10—C1	−153.95 (15)	O2—C19—C20—C30	−62.9 (2)
C11—C9—C10—C5	−174.71 (16)	C18—C19—C20—C30	−177.28 (18)
C8—C9—C10—C5	−44.4 (2)	O2—C19—C20—C21	56.3 (2)
C8—C9—C11—C12	56.7 (2)	C18—C19—C20—C21	−58.1 (2)
C10—C9—C11—C12	−169.56 (16)	C29—C20—C21—C22	−80.1 (3)
C9—C11—C12—C13	−52.8 (2)	C30—C20—C21—C22	158.8 (2)
C11—C12—C13—C18	−178.59 (15)	C19—C20—C21—C22	40.4 (3)
C11—C12—C13—C14	54.06 (19)	C20—C21—C22—C17	−45.1 (3)
C12—C13—C14—C15	−179.09 (15)	C16—C17—C22—C21	−172.49 (18)
C18—C13—C14—C15	54.9 (2)	C28—C17—C22—C21	−46.6 (2)
C12—C13—C14—C27	64.56 (18)	C18—C17—C22—C21	61.9 (2)
C18—C13—C14—C27	−61.45 (19)	C19—O2—C28—C17	0.2 (2)
C12—C13—C14—C8	−58.21 (17)	C16—C17—C28—O2	−149.50 (17)
C18—C13—C14—C8	175.78 (14)	C18—C17—C28—O2	−28.6 (2)
C7—C8—C14—C15	−61.56 (19)	C22—C17—C28—O2	84.4 (2)

### Hydrogen-bond geometry (Å, °)

**Table:**

*D*—H···*A*	*D*—H	H···*A*	*D*···*A*	*D*—H···*A*
O1—H1···O2^i^	0.87 (3)	1.93 (3)	2.782 (2)	164 (3)

Symmetry codes: (i) −*x*+1/2, −*y*+2, *z*+1/2.
